# Impact of tobacco smoking on oral cancer genetics—A next‐generation sequencing perspective

**DOI:** 10.1002/imt2.44

**Published:** 2022-08-04

**Authors:** Vishwas Sharma, Dinesh Kumar, Shravan Kumar, Harpreet Singh, Naveen Sharma, Sanjay Gupta

**Affiliations:** ^1^ Division of Cytopathology ICMR—National Institute of Cancer Prevention and Research Noida Uttar Pradesh India; ^2^ WHO FCTC Global Knowledge Hub on Smokeless Tobacco ICMR‐ National Institute of Cancer Prevention and Research Noida Uttar Pradesh India; ^3^ Indian Council of Medical Research Informatics, Systems & Research Management (ISRM) New Delhi India; ^4^ Biomedical Informatics Division Indian Council of Medical Research New Delhi India

## Abstract

This study identified a total of 28 genetic loci (comprising 31 genes), which were found to be altered in oral cancer patients having a habit of tobacco smoking. Three loci, that is, 17p13.1 (*TP53*), 9p21.3 (*CDKN2A*), and 9q34.3 (*NOTCH1*) were found to be modified and common in three records whereas one locus, that is, 3q26.32 (*PIK3CA*) was found to be modified and common in two records. This study suggests that oral cancer patients should be categorized into different subgroups based on (i) genetic signatures, and (ii) smoking status, then the treatment strategies for each group should be precisely defined.

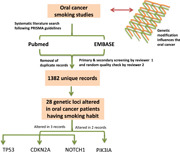

## INTRODUCTION

Tobacco is either smoked or used as smokeless tobacco (tobacco i.e., chewed, sniffed, or sucked) [1, 2]. Smoking and smokeless tobacco both increases the risk of oral cancer (OC). Smoking is collectively defined as a habit of inhalation and exhalation of burned tobacco smoke. It is estimated that >1.3 billion people smoke worldwide [3]. The World Health Organization (WHO) estimates that tobacco causes the death of nearly 8 million people each year, of which smoking contributes to around 4 million [4, 5]. Tobacco smokers have a 5–10 times higher risk of developing OC than nonsmokers, and the risk increases with an increasing frequency of exposure [6–9]. Tobacco smoke contains more than 70 known carcinogenic substances, which can induce genetic changes in cells of the oral cavity [10]. Some of the well‐documented carcinogenic chemicals in tobacco smoke are polycyclic aromatic hydrocarbons (PAHs), *N*‐nitrosamines, aromatic amines, and certain volatile organic agents whose metabolites are elevated in the urine of smokers when compared to those of nonsmokers [11]. PAH activation by cytochrome P450 (drug‐metabolizing enzymes) and aldoketo reductases (AKRs) produce highly reactive carcinogenic electrophiles, which can cause cellular transformation [12, 13]. The activated PAHs lead G to T transversions in genomic DNA [13, 14]. The mechanism through which carcinogenic substances in tobacco contribute to oral carcinogenesis involves three major steps, that is, (1) exposure to the carcinogenic substances, (2) formation of covalent bonds between the carcinogens and DNA to form an adduct, and (3) resultant accumulation of permanent somatic mutations at various genetic loci in the genome, which leads to clonal outgrowth, and an accumulation of additional mutations, leading to the development of OC [11].

As genetic changes play a key role in the initiation and progression of OC, in recent years, various studies have utilized next‐generation sequencing (NGS) to study the changes at genomic (DNA) and transcriptomic (RNA) level [7, 9, 15, 16]. In NGS technology,  massively parallel sequencing of millions of small fragments of DNA is quickly performed. Unlike the classical methods like Sanger sequencing, Real‐time polymerase chain reaction (PCR), Microarray, and SNP array, the NGS has higher specificity, sensitivity, and genomic coverage, and hence has emerged as a better choice to study the impact of smoking on OC genetics [17]. Hitherto, based on the systematic literature search (Supporting Information: Table S1), we could not find any review that could provide information on the genetic changes associated with OC in smokers, identified using an NGS platform. This review highlights the modified genetic loci in OC patients with smoking from the relevant records utilizing NGS technology. The information obtained from this study could be beneficial for future research on targeted therapy in OC.

## RESULTS

**Figure 1:**
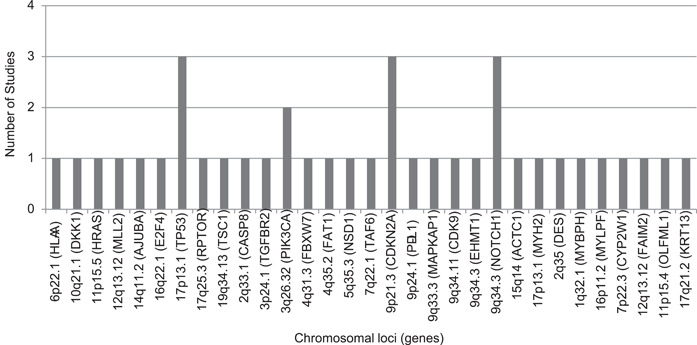
Modified chromosomal loci with the number of records.

**Figure 2:**
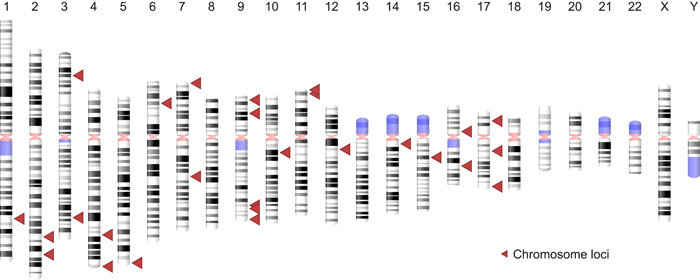
Mapped modified genetic loci on oral cancer in patients who smoke tobacco. Information on genetic loci with their encompassing gene is depicted in Supporting Information: Table S2.

Of the seven records finally included for analysis, a total of 28 altered genetic loci (comprising 31 genes) were found in OC patients who smoke (Figure 1 and Supporting Information: Table S2). Of these 28 loci, three loci, that is, 17p13.1 (*TP53*), 9p21.3 (*CDKN2A*), and 9q34.3 (*NOTCH1*) were found to be altered in three records whereas one locus, that is, 3q26.32 (*PIK3CA*) was found to be altered in two records (Supporting Information: Table S2). For better representation, all the four loci found to be common and modified in ≥2 records were mapped on human chromosomes using the Genome Decoration tool (https://www.ncbi.nlm.nih.gov/genome/tools/gdp) (Figure 2). The known function of 28 genetic loci encompassing 31 genes is detailed in Supporting Information: Table S2.

## DISCUSSION

Smoking has been shown to increase the risk of OC, which tends to increase with the frequency of intake with time [18]. The mechanism through which smoking initiates and progresses the development of OC is complex, but it is quite evident that smoking alters both the genomic (DNA level) and transcriptomic (RNA level) architecture of OC patients. Familiarity with the variabilities at both levels will likely assist in (i) understanding the complex biology of OC (ii) defining the important regions in the genome and/or transcriptome that could be targeted for the discovery of new therapeutic modalities, and (iii) refining treatment strategy for the patients with OC.

This study found four hotspots, that is, 17p13.1 (*TP53*), 9p21.3 (*CDKN2A*), 9q34.3 (*NOTCH1*), and 3q26.32 (*PIK3CA*) with 24 other altered genetic loci in OC patients with smoking habit. All identified genetic loci need further in‐depth validation through fine‐mapping studies to dissect the association signals and functional studies to define and validate the role of these regions in oral carcinogenesis.

*TP53* or *p53* is a tumor suppressor gene that acts as a key regulator of a cell's genomic stability, function, and homeostasis [19]. Alteration in genomic stability, cellular function, and homeostasis can be attributed to cancer progression. *TP53* is a key factor for normal cellular growth and differentiation. Mutation or inactivation of *TP53* has been associated with >50% of human cancers [20]. This study confirms that TP53 is mutated, and altered transcript expression of TP53 has been observed in OC tissue while compared with paired blood DNA profiling from the same participant in different records in smokers. TP53 is mutated in more than 70% of oral squamous cell carcinoma (OSCC) [21] and is associated with reduced survival of OSCC patients [22]. Different types of p53 mutations have been observed in the early stages of OC development [21, 22]. Three novel mutations out of 51, that is, frameshift deletion in exon 4; G > T transversion at codon 117 in exon 4 and G > A transition at codon 319 in exon 9 have been reported in OC tissues [21]. Moreover, Ragos et al. reported that missense and truncating mutations are linked with malignant cell proliferation, resistance to chemotherapeutic regimens, and increased invasion [22]. All of these biochemical and histological factors contribute to poor prognosis (decreased drug response rates), especially in missense mutation carriers. Further, a study on 345 OSCC patients reported that the carriage of missense mutations in the TP53 DNA binding domain (DBD missense mutations) is linked with decreased disease‐specific survival (DSS) as compared with wild‐type TP53 [23]. Genetic variability in the p53 gene leads to modifications in the proapoptotic balance causing drug resistance [24].

The gene *CDKN2A* encodes the cell‐cycle inhibitor protein p16 [25]. A study performed on 73 OSCC tumor patients found downregulated expression of CDKN2A to be associated with OC recurrence and the authors suggested that this could be used as a biomarker for cancer prognosis [26]. Our study revealed an association between altered gene expression and mutation of *CDKN2A* in OC. Mutated *CDKN2A* has been reported in OC paired samples. Riese et al. sequenced exon 2 of *CDKN2A* and reported transversion G to T at position 322 (Asp108Tyr) and another well‐defined polymorphism at Ala148Thr in 5 out of 70 primary head and neck cancers obtained from oropharynx, hypopharynx, larynx, and oral cavity [27]. Moreover, epigenetic inactivation of *CDKN2A* has also been reported in 16 out of 55 cases of OSCC. Deletion/loss of function is associated with recurrent cases attributing its role to malignant transformations. The *CDKN2A* locus is prone to homozygous deletion [28] and it has been reported that the majority of oral tongue squamous cell carcinomas did not show elevated *CDKN2A* expression [29].

NOTCH family genes have been studied widely for cancer development, progression, and metastasis. A study on 144 samples from China found mutations in 54% of primary OSCC and 60% of lesions in patients with oral potentially malignant disorders, clearly depicting that mutation in *NOTCH1* promotes distinct oral tumorigenesis [30]. *NOTCH1* was found to be upregulated in tongue carcinoma tissues, and to enhance tongue cancer cell proliferation and invasion [31]. Animal studies on rats have also demonstrated membranous *NOTCH1* expression to be linked with the severity of dysplasia and the growth of OSCC [32].

The phosphatidylinositol 3‐kinase (PI3K) comprises a catalytic subunit (p110a) encoded by oncogene *PIK3CA*. The gene *PIK3CA* is located on chromosome locus 3q26, which is often over‐amplified in OSCC [33, 34]. Highly frequent mutations in this gene were first reported by Samuel et al. [35], which vary from 0% to 21% [36, 37]. A study from India found polymorphism rs17849071 G/T in this gene to be inversely linked to OC [38]. A significant correlation between somatic mutations and disease stage was also reported [39]. Elevated expression of PIK3CA has been observed at the transcript level in OSCC [40], yet the association of genetic variations with the expression level has not been observed [41, 42]. Most probably, it could be due to the late occurrence of somatic mutations in the development of OSCC and tumor progression occurring via the PI3K–AKT signaling pathway [39].

Although the data in the current review is heterogeneous, due to the limited number of records available, smoking was taken as an umbrella without segregating it into its subtypes. For a more robust interpretation, a larger number of records at a subtype level of smoking status are required from different geographical domains. The limited number of records found during a comprehensive literature search highlights the importance of this area of research in smoking‐associated OC. Apart from smoking, the use of smokeless tobacco and areca nut products is also very frequent in South East Asian countries, especially in India. All these products also increase the risk of OC [43]. Studying the impact of these factors on OC genomics will help in understanding the root cause of the OC.

## CONCLUSIONS

As the cost of NGS sequencing is decreasing, it is quite likely that in the next few years more records will be available from a similar perspective that will provide a more profound understanding of the smoking‐related genetic modifications in OC patients. We have discussed genetic loci common in ≥2 records; however, additional 24 genetic loci also contributes to the etiology of OC. This study suggests that OC patients should be categorized into different subgroups based on (i) genetic signatures, (ii) smoking status, (iii) smokeless tobacco, (iv) areca nut, (v) alcohol, (vi) HPV, and so on and the treatment strategies for each group should be precisely defined. Besides, this study would also be beneficial to the diagnostic/pharmaceutical industry in developing new targeted therapies for smoking‐associated OC. Policymakers may use these findings as evidence to help in reframing tobacco control policies for smoking. As smoking modifies the genome and transcriptome of OC patients, there is a clear message that there should be conscious efforts to quit its use as well as other products, such as smokeless tobacco, areca nut, and alcohol.

## METHODS

**Figure 3:**
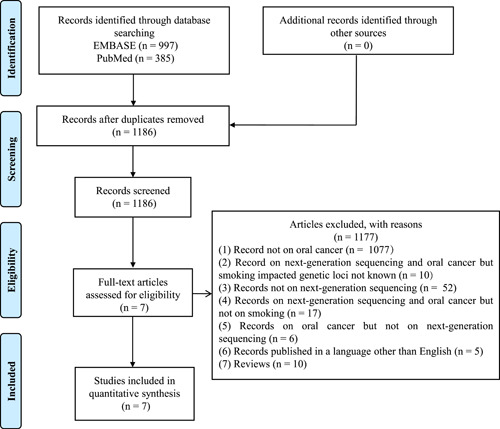
Flow chart describing the included/excluded literature.

A systematic literature search was performed following PRISMA guidelines, to find the records that utilized NGS to study the impact of smoking on OC genetic loci [44, 45, 46] (Figure 3, and Supporting Information: Tables S1 and S3). Briefly, the search was performed from the inception date to May 19, 2020 on EMBASE and the 8th of July 2019 on PubMed. Through EMBASE a total of 997 records were obtained and PubMed provided 385 records, hence a total of 1382 records were retrieved. After removing duplicates, 1186 records were obtained. In addition, freewheeling searches were also performed to retrieve additional records, but no record was obtained through this search. One reviewer (V. S.) screened all titles and abstracts and relevant full text of records. A second reviewer (D. K. S) performed a quality check of a random selection of 10% of titles, abstracts, and full text of records. Discrepancies were resolved; when a consensus was not reached, a third reviewer was consulted. Relevant data from all included records were extracted by a single reviewer, using a predefined extraction grid, which was subsequently validated by an independent reviewer. After removing duplicates, a total of 1186 records were obtained. The detailed methodology and search terms used are presented in Supporting Information: Table S1.

### Inclusion criteria

Records that utilized NGS and highlighted the modified genetic loci (genes) in OC patients with smoking were considered {n = 7} (Table S4).

### Exclusion criteria

(1) Records not on oral cancer (*n* = 1077).
(2) Records on next‐generation sequencing and OC but smoking impacted genetic loci not known (*n* = 10).
(3) Records not on next‐generation sequencing (*n* = 52).
(4) Records on next‐generation sequencing and oral cancer but not on smoking (*n* = 17).
(5) Records on oral cancer but not on next‐generation sequencing (*n* = 6).
(6) Records published in a language other than English (*n* = 5).
(7) Reviews (*n* = 10).

## AUTHOR CONTRIBUTIONS

Vishwas Sharma and Dinesh Kumar were involved in designing the study, collecting information, analyzing and interpreting of data, and writing the first draft of the manuscript. Shravan Kumar screened the record. Harpreet Singh, and Naveen Sharma reviewed the manuscript. Sanjay Gupta lead the group and reviewed the final draft.

## CONFLICT OF INTEREST

The authors declare no conflict of interest.

## Supporting information

Supporting information.

## Data Availability

The data used in the analysis are freely available on the SRA data repository (https://www.ncbi.nlm.nih.gov/sra). No new data and scripts were used in this paper. Supplementary materials (figures, tables, scripts, graphical abstract, slides, videos, Chinese translated version, and update materials) may be found in the online DOI or iMeta Science http://www.imeta.science/.
